# The host transcriptional response to superinfection by influenza A virus and *Streptococcus pneumoniae*

**DOI:** 10.1128/msystems.01048-23

**Published:** 2024-03-06

**Authors:** Ofir Cohn, Gal Yankovitz, Michal Mandelboim, Naama Peshes-Yaloz, Rachel Brandes, Eran Bacharach, Irit Gat-Viks

**Affiliations:** 1 The Shmunis School of Biomedicine and Cancer Research, George S. Wise Faculty of Life Sciences, Tel Aviv University, Tel Aviv, Israel; 2 Central Virology Laboratory, Ministry of Health, Chaim Sheba Medical Center, Ramat Gan, Israel; 3 Department of Epidemiology and Preventive Medicine, Sackler Faculty of Medicine, Tel Aviv University, Tel Aviv, Israel; Princeton University, USA

**Keywords:** superinfection, influenza, *Streptococcus pneumoniae*, RNA sequencing, host resistance, system biology

## Abstract

Secondary bacterial challenges during influenza virus infection “superinfection”) cause excessive mortality and hospitalization. Here, we present a longitudinal study of bulk gene expression changes in murine lungs during superinfection, with an initial influenza A virus infection and a subsequent *Streptococcus pneumoniae* infection. In addition to the well-characterized impairment of the host response, we identified superinfection-specific alterations in the global transcriptional program that are linked to the host’s ability to resist the pathogens. Particularly, whereas superinfected mice manifested an excessive rapid induction of the resistance-to-infection program, there was a substantial tissue-level rewiring of this program: upon superinfection, interferon-regulated genes were switched from positive to negative correlations with the host’s resistance state, whereas genes of fatty acid metabolism switched from negative to positive correlations with resistance states. Thus, the transcriptional resistance state in superinfection is reprogrammed toward repressed interferon signaling and induced fatty acid metabolism. Our findings suggest new insights into a tissue-level remodeling of the host defense upon superinfection, providing promising targets for future therapeutic interventions.

**IMPORTANCE:**

Secondary bacterial infections are the most frequent complications during influenza A virus (IAV) pandemic outbreaks, contributing to excessive morbidity and mortality in the human population. Most IAV-related deaths are attributed to *Streptococcus pneumoniae* (SP) infections, which usually begin within the first week of IAV infection in the respiratory tracts. Here, we focused on longitudinal transcriptional responses during a superinfection model consisting of an SP infection that follows an initial IAV infection, comparing superinfection to an IAV-only infection, an SP-only infection, and control treatments. Our longitudinal data allowed a fine analysis of gene expression changes during superinfection. For instance, we found that superinfected mice exhibited rapid gene expression induction or reduction within the first 12 h after encountering the second pathogen. Cell proliferation and immune response activation processes were upregulated, while endothelial processes, vasculogenesis, and angiogenesis were downregulated, providing promising targets for future therapeutic interventions. We further analyzed the longitudinal transcriptional responses in the context of a previously defined spectrum of the host’s resistance state, revealing superinfection-specific reprogramming of resistance states, such as reprogramming of fatty acid metabolism and interferon signaling. The reprogrammed functions are compelling new targets for switching the pathogenic superinfection state into a single-infection state.

## INTRODUCTION

Since the dawn of human history, infectious diseases have placed a significant burden on public health and the global economy (1), with respiratory infections constituting a leading cause of morbidity and mortality worldwide, particularly in immunocompromised subpopulations, such as young children and older adults (2). Among respiratory viral pathogens, the influenza A virus (IAV) poses a continual threat to global health as it is responsible for seasonal outbreaks and occasional pandemics in humans, contributing to 3–5 million cases of severe disease and 290,000–650,000 deaths annually (3), with an increased mortality rate during pandemic years (4). This enhanced pathogenesis is associated with secondary bacterial pneumonia, attributed to 40%–95% of IAV-related mortality during past pandemics (5). *Streptococcus pneumoniae* (SP) is a commonly identified bacterium in IAV pandemics and a prominent etiological agent of secondary bacterial pneumonia. As suggested by clinical and autopsy examinations, during the “Spanish” IAV pandemic in 1918–1919, more than 95% of the deaths (~50 million) were complicated by bacterial infections, most commonly by SP (6, 7). The combination of viral infection (e.g., IAV) and secondary infection by a different microbe is called “superinfection.”

Several physical and immunological mechanisms explain the increased permissiveness of IAV-infected lungs to subsequent bacterial infections. Particularly, various studies highlighted virus-induced damage of the respiratory epithelium as a primary factor for increased bacterial adherence and replication, resulting in a loss of lung repair processes and impaired function (8–11). In addition, multiple studies have revealed IAV-infection-dependent reduction in innate immune activity, such as depletion or dysfunction of macrophages and neutrophils that are essential for early bacterial clearance (12, 13). This dysfunction, in turn, leads to elevated production of cytokines and chemokines (14–16) that subsequently affect other immune cell types (e.g., T cells and monocytes) (17, 18). Global gene expression analyses further allowed an unbiased genome-scale discovery of candidate mechanisms, such as reduced lung epithelial cell proliferation, reduced tissue repair processes, and dysregulation of apoptosis (9, 19).

In a recent study, we have defined a global molecular program for the host’s resistance against pathogens (20). This resistance program (i) determines the global transcriptional state in a large variety of infections; (ii) exists at the molecular level in both immune and non-immune cells; (iii) explains a large part of the inter-individual variation in response to infection, both in human and mouse; and (iv) can be used to predict future susceptibility to infections. Although the resistance program has been characterized during a large variety of infections, it has not been investigated during superinfections.

Here, we analyzed longitudinal transcriptional responses during superinfection in the context of the host’s resistance state. We focused on a superinfection model consisting of an SP infection that follows an initial IAV infection, comparing superinfection to IAV-only infection, SP-only infection, and control (PBS) treatments. We report that superinfected mice manifest an excessive rapid induction of the host-resistance program, starting only a few hours after the secondary bacterial challenge. Furthermore, by accounting for the global resistance state in the lungs, we revealed a novel tissue-level reprogramming of the resistance program (namely, a change in gene expression relative to the resistance state), particularly for interferon signaling and fatty acid metabolism genes. Overall, our study identified a remodeling mode of the host defense, operating in superinfection rather than in single-pathogen infection.

## RESULTS

### The IAV/SP superinfection is associated with a severe disease course

**Fig 1:**
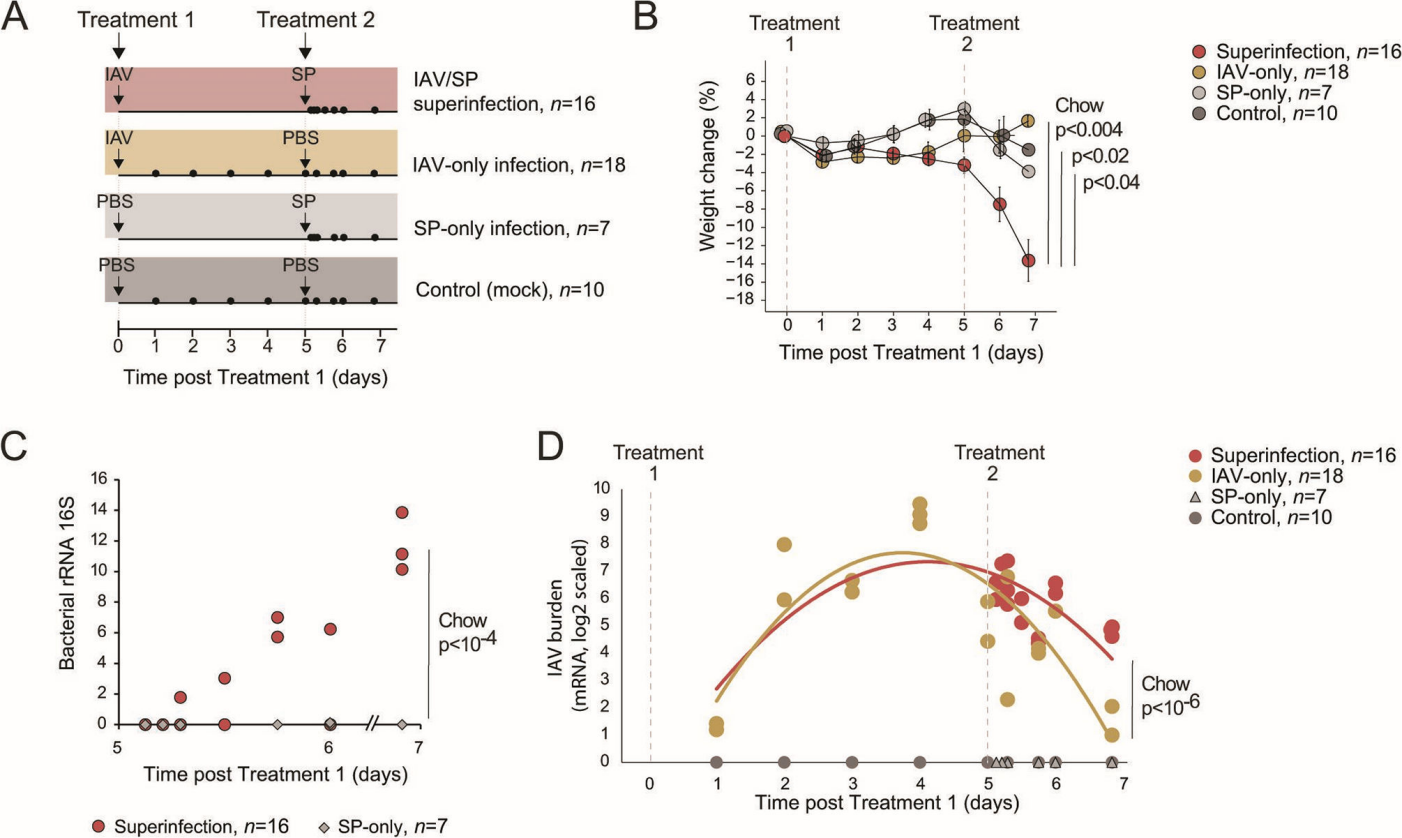
High disease severity in superinfection with IAV and SP. (**A**) Schematics of the study design and timelines for IAV/SP superinfection (*n* = 16), IAV-only infection (*n* = 18), SP-only infection (*n* = 7), and control (mock, *n* = 10) treatments (Table S1). Each individual is represented as a circle located according to the time of its analysis. (**B**) Mean percent weight loss from baseline ± standard error for each treatment at the indicated time points. Chow’s *P*-values compare superinfection vs each other group using all weighting time points after Treatment 2 (*n* = 12, 7, 5, and 5 for superinfection, IAV-only, SP-only, and control groups, respectively). (**C**) Quantification by qRT-PCR of bacterial 16S rRNA copies in the lungs of superinfected and SP-only infected mice at indicated days after Treatment 1. Individual samples are shown as dots. The Chow *P*-value compares superinfection vs SP-only using all 16S rRNA measurements (*n* = 16 and 7 for superinfection and SP-only animals, respectively). (**D**) Viral burden in the lungs, based on the quantification of viral mRNA levels (log2 scaled). Individual samples are shown as dots. Curves: polynomial regression fitting (order 2); superinfection/IAV-only in red/mustard lines. The Chow *P*-value compares superinfection vs IAV-only using all viral mRNA measurements after Treatment 2 (time point > 5 days; *n* = 16 in the superinfection group and *n* = 7 in the IAV-only group).

To investigate superinfection, we followed a model (14, 21) in which mice were exposed to an SP treatment 5 days after the initial IAV infection (*n* = 16 mice during days 5–7 post-Treatment 1) (Fig. 1A). We compared the IAV/SP superinfection to three control treatments: IAV-only infection (IAV on day 0, PBS on day 5, *n* = 18 mice during days 1–7 post-Treatment 1), SP-only infection (PBS on day 0, SP on day 5, *n* = 7 mice during days 5–7 post-Treatment 1), and a mock-infection control (PBS on days 0 and 5, *n* = 10 mice during days 1–7 post-Treatment 1) (Fig. 1A; Table S1; see Materials and Methods). Several lines of evidence demonstrated the enhanced severity of the superinfection compared to the other treatments. First, superinfected mice showed increased gradual weight loss compared to each of the other groups [Fig. 1B; Chow test *P* < 0.004, 0.04, and 0.02 for superinfection against the IAV-only, SP-only, and control groups, respectively, using the time points after Treatment 2 (*n* = 12, 7, 5, and 5, respectively, Table S1)]. Second, superinfected mice had a higher lung bacterial load than those infected only with bacteria [Fig. 1C; superinfection vs SP-only Chow test *P* < 10^−4^ (*n* = 16 and 7, respectively, Table S1); bacterial load was measured by quantitative RT-PCR (qRT-PCR) of 16S rRNA, see Materials and Methods]. Third, we quantified the viral burden in the lungs by measuring lung transcriptomes that captured both the host and the viral mRNA. The viral burden in superinfected mice was similar to or higher than the viral burden in the IAV-only group [Fig. 1D; Chow test *P* < 10^−6^ for the comparison of viral burden after Treatment 2 between the superinfection and the IAV-only groups (*n* = 16 and 7, respectively, Table S1)]. Thus, consistent with previous studies (22, 23), IAV/SP superinfection has a substantial severity compared to IAV-only and SP-only infections.

### The IAV/SP superinfection is associated with massive induction and repression of gene expression

**Fig 2:**
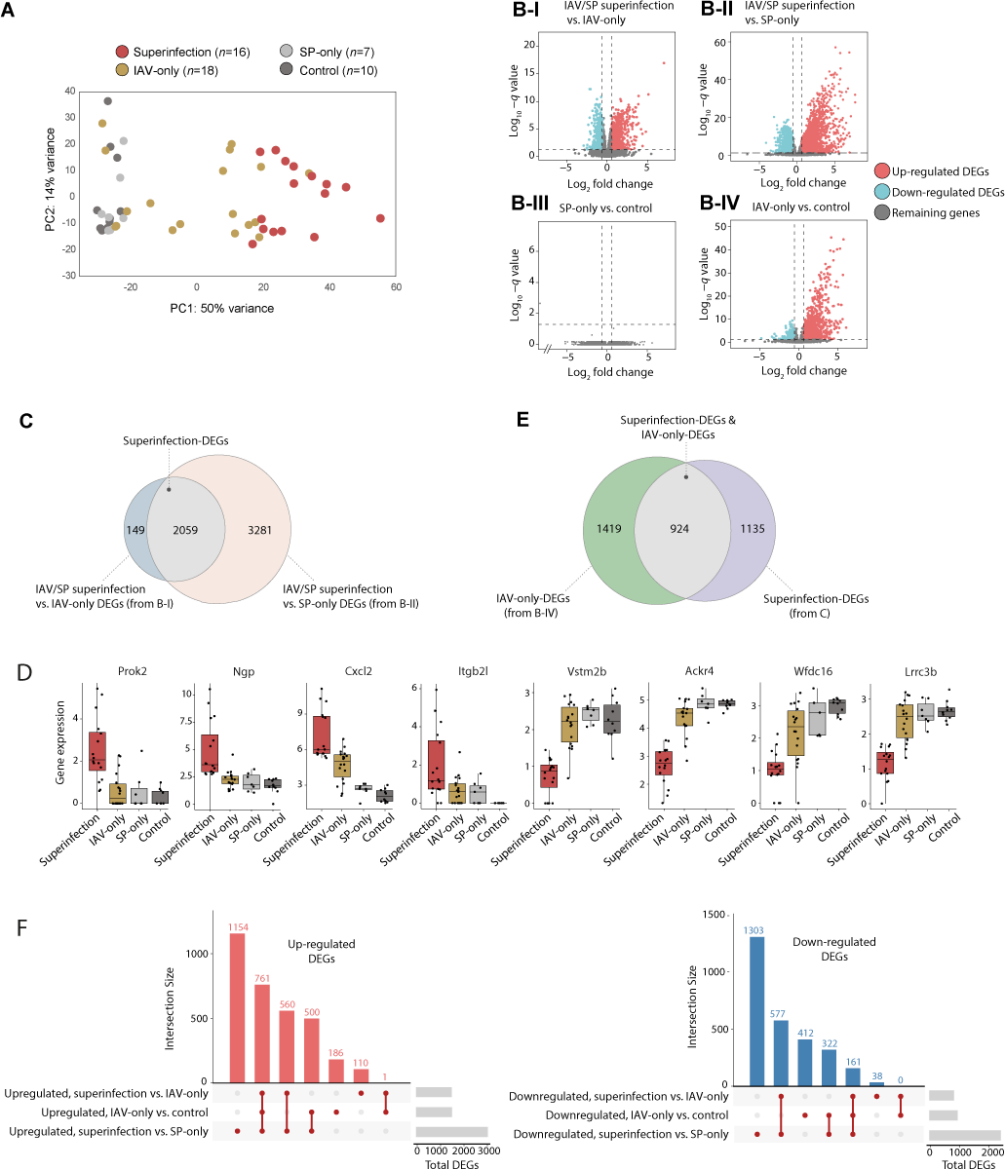
Massive superinfection-specific induction and repression of genes. (**A**) IAV/SP superinfection is associated with a global change in the overall gene expression state. Principal component analysis of transcriptome profiles of mice from the four treatment groups. For each mouse (a circle), its treatment group is color coded. (**B**) Volcano plots indicating the differentially expressed genes (DEGs) of the compared treatment groups (each dot represents a single gene). DEGs are indicated in blue (downregulated) or red (upregulated). The remaining genes are indicated in gray. The compared groups consist of all superinfection mice (*n* = 16), SP-only mice (*n* = 7), control mice (*n* = 10), and the IAV-only mice at 2–days post-Treatment 1 (*n* = 14). (**C**) Venn diagram displaying the intersections among the DEGs of the indicated comparisons from panel B. (**D**) Selected superinfection DEGs. Presented are four upregulated (left) and four downregulated (right) superinfection DEGs. Individual samples are shown as dots. All mice are included (*n* = 16, 18, 7, and 10 for superinfection, IAV only, SP only, and controls). (**E**) Venn diagram displaying the intersection between two sets: superinfection DEGs (as in panel C) and IAV-only DEGs (from panel B-IV). (**F**) UpSet plot visualizing the intersections of three sets of DEGs: superinfection vs IAV only, superinfection vs SP only, and IAV only vs control. Left/right: each gene set includes only the upregulated/downregulated genes. In all plots, DEGs are genes with *q*-value < 0.05 and FC > 1.5, listed in Table S2.

Given the limited knowledge about the factors responsible for the increased host vulnerability upon superinfection, we aimed to identify host transcriptional changes manifested explicitly in the secondary infection. As an initial indication, we observed that the overall transcriptional state is distinct in IAV/SP superinfection compared to the other treatments (Fig. 2A; Fig. S1A). Particularly, we observed a clear separation along the first principal component (PC1) axis between the IAV/SP superinfection treatment and the other treatments.

We propose that superinfection-specific genes are those that are differentially expressed between the response to the IAV + SP treatments (“superinfection” samples, after Treatment 2 with SP, *n* = 16) and the response to a single treatment: either an IAV-only treatment (the IAV-only group, time 2–6 days post-Treatment 1, *n* = 14) or an SP-only treatment (the SP-only group, after Treatment 2 with SP, *n* = 7) (see details in Table S1 and Materials and Methods). In the comparison of IAV/SP superinfection to IAV-only samples, we detected 2,208 differentially expressed genes (DEGs), including 1,432 genes (65%) that were significantly upregulated and 776 genes (35%) that were significantly downregulated [using *q* value < 0.05 and fold change (FC) > 1.5 cutoffs; Fig. 2B-I; Table S2; see Materials and Methods]. Importantly, 93% (2,059) of the genes that were differentially expressed in superinfection compared to IAV-only infection were also differentially expressed in superinfection compared to SP-only infection [using *q* value < 0.05 and FC > 1.5 in Fig. 2B-II; Table S2; hypergeometric (HG) *P* ≪ 10^−100^ for the overlap in Fig. 2C). We refer to these 2,059 genes, which are differentially expressed between superinfection and a single (IAV or SP) infection, as “superinfection DEGs.” The superinfection DEGs include 1,321 upregulated and 738 downregulated genes (Table S2; exemplified in Fig. 2D). The massive number of genes primarily respond to superinfection— rather than IAV-only or SP-only infections—highlights a substantial transcriptional response unique to secondary infection.

Next, we asked whether these superinfection DEGs are differentially expressed when a single infection is compared to the controls (a mock infection). In the following, we first describe the SP-only experiment compared to the control and then the IAV-only experiment compared to the control (*n* = 7, 14, and 10 for SP only, IAV only, and control, respectively, see Materials and Methods). We found only one DEG for SP-only treatment compared to control *q* value ≤ 0.05 and FC > 1.5, denoted “SP-only DEG,” Fig. 2B-III; Table S2), implying that the superinfection response is not observed in the SP-only treatment. In contrast, there was an extensive change in expression following IAV-only treatment compared to control treatment (2,343 DEGs, *q* value ≤ 0.05 and FC > 1.5, denoted “IAV-only DEGs”) (Fig. 2B-IV; Table S2). Many superinfection DEGs were also IAV-only DEGs (924 of 2,059 genes, HG *P* ≪ 10^−100^; Fig. 2E). Of these 924 genes, 922 (>99%) responded in the same directions as superinfection DEGs and IAV-only DEGs (Table S2). Thus, much of the superinfection response is also observed in the IAV-only response but not in the SP-only response.

Several lines of evidence provide additional support for these findings. First, there is a reproducibility of the IAV effect between different groups of mice (either with or without a subsequent SP treatment, Pearson’s *P* < 10^−100^; Fig. S1B). Second, the effect sizes (log_2_ fold changes) of the SP-only-vs-control comparison are substantially lower than the effect sizes of the other comparisons (paired *t*-test *P* < 10^−138^ in all cases; Fig. S1C), consistent with the rarity of the SP-only DEGs (Fig. 2B-III). Third, clustering of the top-varying genes (Fig. S2) supports various observations: there is an amplification of response in superinfection (clusters 1–3); the superinfection response is sometimes observed in the IAV-only response (e.g., cluster 1), but it is not commonly observed in the SP-only response (clusters 1–3). Finally, we found a clear difference between upregulated and downregulated superinfection DEGs: among 1,321 upregulated superinfection DEGs, 761 genes (57%) were also upregulated IAV-only DEGs, whereas among the 738 downregulated superinfection DEGs, only 161 (21%) were also downregulated in the IAV-only treatment (*P* < 10^−5^, χ^2^ test; Fig. 2F). Thus, the extensive downregulation is a unique property of superinfection when compared to IAV-only infection. In contrast, part of the upregulation in superinfection amplifies the initial transcriptional response to IAV infection.

**Fig 3:**
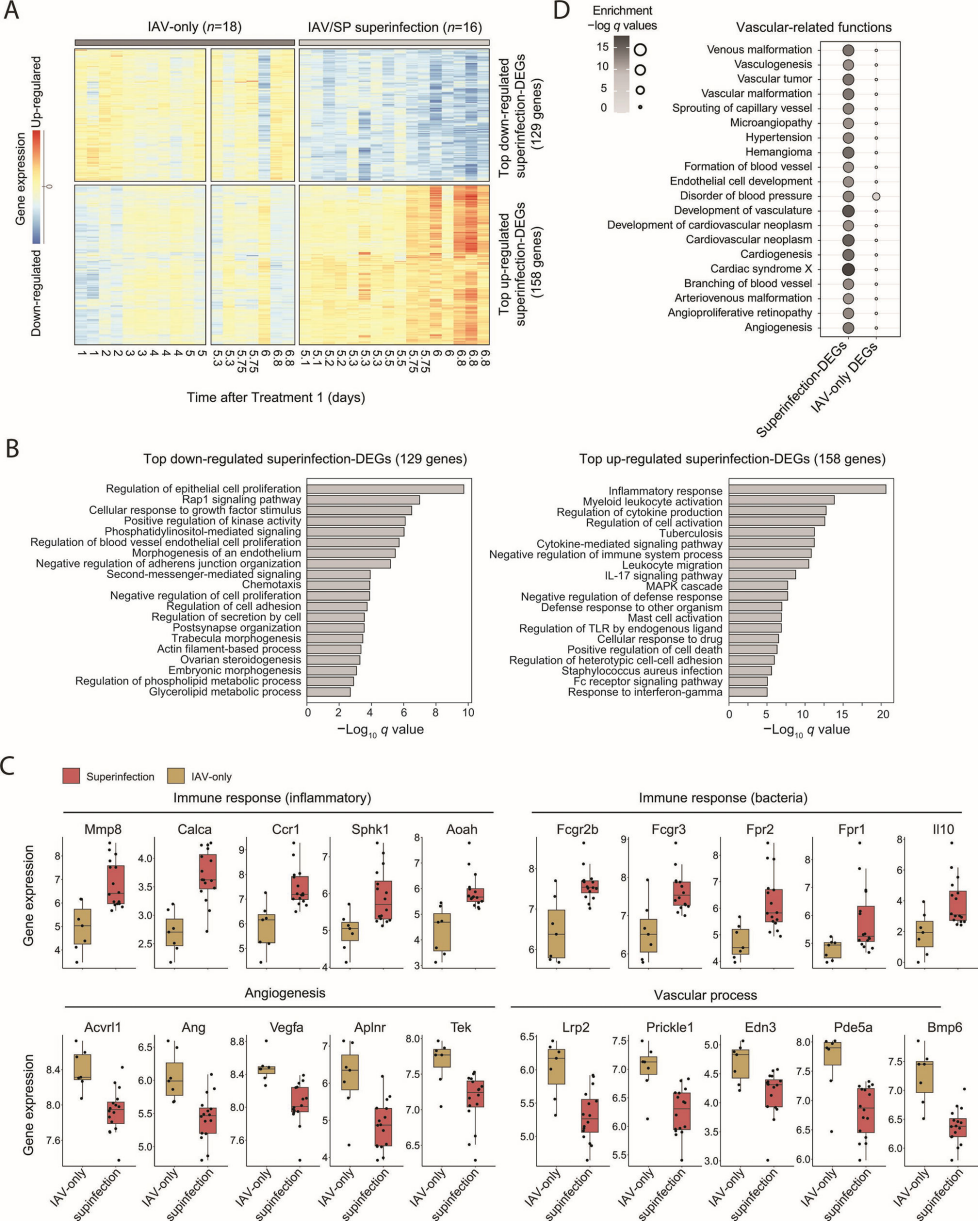
Functional characterization of the top superinfection DEGs. (**A**) Heatmap showing gene expression of top 287 superinfection DEGs from Fig. 2. Columns represent a sample (specific treatment and time point), and rows represent genes. The red and blue gradients indicate up- and downregulated gene expression, respectively, relative to untreated mice. (**B**) Gene ontology enrichment analysis of the downregulated (left) and upregulated (right) top superinfection DEGs from panel A. Presented are FDR-adjusted -log *q*-values (HG test). (**C**) Boxplots of exemplified genes from panel A, providing gene expression (log2 scaled) of vascular, angiogenesis, and immune-related genes in the IAV-only (mustard) and the IAV/SP superinfection groups (red). Individual samples are shown as dots, including all mice after Treatment 2 (IAV only: *n* = 7 and superinfection: *n* = 16; Table S2). (**D**) A bubble plot demonstrating enrichments in the superinfection DEGs (2,509 genes, left) and IAV-only DEGs (2,343 genes, right) for various vascular-related functions from the Ingenuity Knowledge Base annotation. The gene sets of DEGs are from Fig. 2C and E. Bubble size and color scale represent the FDR-adjusted HG test *q*-values.

We next focused on the superinfection DEGs for which the differences between superinfection and single infections are maximized (158 upregulated and 129 downregulated superinfection DEGs for which IAV/SP superinfection vs IAV-only *q* value < 10^−5^, specified in Table S2). As shown in Fig. 3A, the top downregulated and upregulated superinfection DEGs represent a rapid change in the transcript levels within several hours of post-secondary bacterial infection. Functional analysis revealed that the downregulated superinfection DEGs are involved in the regulation of epithelial cell proliferation (*P* < 10^−9^, HG test), consistent with previous findings (9, 19). Interestingly, we further revealed that the downregulated superinfection DEGs are involved in angiogenesis and vascular-associated terms, such as “regulation of blood vessel endothelial cell proliferation” (*P* < 10^−5^, HG test) and “morphogenesis of an endothelium” (*P* < 10^−5^, HG test) (Fig. 3B; see exemplified genes in Fig. 3C). In agreement, vascular-related functions from the Ingenuity annotation are mainly enriched in the superinfection DEGs compared to the enrichment in the IAV-only DEGs (Fig. 3D). The top upregulated superinfection DEGs are involved in immune responses, such as inflammation (HG test *P* < 10^−20^) and cytokine production (HG test *P* < 10^−12^)—e.g., *Il10, Fcgr3,* and *Ccr1* (Fig. 3B and C). Therefore, our findings highlight endothelial and vascular dysregulation, which could be related to the documented increased risk of barrier disruption, pulmonary thrombosis, and excessive vascular leak (24, 25). Of note, whereas some of the top superinfection DEGs are well established [e.g., *Cxcl2* (26)], many other superinfection DEGs are not yet reported, such as *Aoah* and *Aplnr* (Fig. 3C).

### Reprogramming of the host’s resistance program during superinfection

We next sought to investigate the molecular state of the host’s program of resistance against invading pathogens, building on a predefined transcriptional signature for this program’s activity (20) (see Materials and Methods). This signature allowed us to calculate the “resistance level” in the lungs of each mouse using a deconvolution approach (see Materials and Methods). Previous evaluation of the resistance levels across cohorts showed the accuracy of this program during single infection (viral or bacterial infection), interpreted the function of this program in resistance against invading pathogens, and demonstrated that this program is part of a generic molecular response in both immune and non-immune cell types (20) (see Materials and Methods). In additional evaluation, we found high relevance of this program in the context of superinfection: the resistance level explains high percentages of the variation in gene expression across individuals, both in IAV infection and in IAV/SP superinfection (see Materials and Methods; Fig. S3A), and the model explains high percentages of the variation among genes within each individual, in each of the experimental groups (see Materials and Methods; Fig. S3B). For example, for 50% of the individuals in the superinfection group, the percentage of inter-gene variance explained by the model is above 25% (Fig. S3B). In fact, we observe higher percentages of explained inter-gene variation in superinfected individuals compared to individuals of the SP-only and IAV-only groups (Wilcoxon *P*-value < 10^−8^ and 10^−3^, respectively; Fig. S3B, left). Encouraged by these findings, we used the resistance program to analyze superinfection. Three samples were outliers of the resistance model and were therefore excluded from further analysis (Fig. S4; see Materials and Methods).

**Fig 4:**
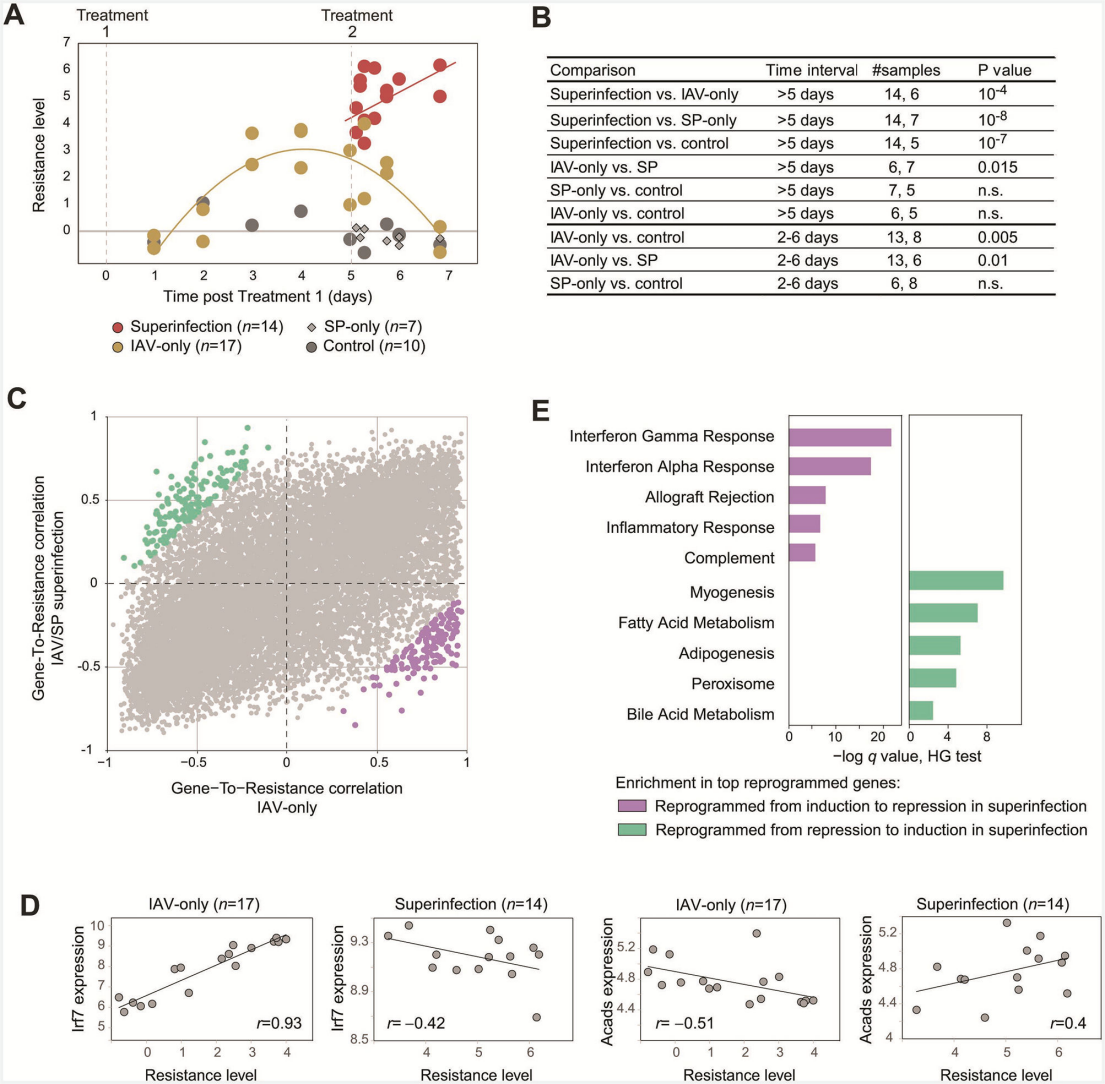
Tissue-level reprogramming of the resistance program in superinfection. (**A**) The host resistance levels (*y*-axis) for each individual (a dot) in each time point (*x*-axis). After excluding three outliers (see Materials and Methods), *n* = 14, 17, 7, and 10 for superinfection, IAV-only, SP-only, and control groups, respectively. (**B**) Comparisons of resistance levels. The table reports Chow-test *P*-values for the comparison of resistance levels (column 4) between two treatment groups (column 1). The time interval of the comparison and the number of compared individuals in this time interval are reported in columns 2 and 3, respectively. (**C**) An overview of superinfection-specific changes in gene-to-resistance correlations. For each gene (a dot), the gene-to-resistance correlations are defined across IAV-only mice (*n* = 17, *x*-axis) and superinfected mice (*n* = 14, *y*-axis). Highlighted are 300 genes with top changes in their resistance correlations between the IAV only and superinfected (150 genes in each direction), referred to as “reprogrammed genes” and color coded by their direction of reprogramming. (**D**) Demonstration of selected genes in which the correlations with resistance are altered between the IAV-only and the IAV/SP-superinfection groups. In each scatter plot, shown are the relation between resistance levels (*x*-axis) and the expression of a gene (*Irf7* or *Acads*, *y*-axis) across individuals (dots) from a specific treatment group (indicated on top). The numbers of mice are indicated on top (see details for excluding three outliers in Materials and Methods). (**E**) Enrichment analysis of the top-reprogrammed genes. Gene sets are as defined in panel C. Presented are FDR-adjusted HG test *q*-values.

Given the calculated resistance levels, we found that the general resistance level in response to superinfection is consistent with previous observations (20): (i) resistance was upregulated in response to moderate and severe infections (Fig. 4A and B), and (ii) superinfection was marked by higher pathogen and higher resistance levels compared to a single infection (Fig. 4A and B and Fig. 1C and D), in agreement with the general understanding that the resistance level is coordinated with the pathogen load.

We next analyzed the correlation of each expressed gene with the level of resistance across individuals. The correlation was calculated across individuals using a separate analysis of the IAV-only (*n* = 17) and the superinfection (*n* = 14) groups, allowing us to ask whether and how these correlations are altered in superinfection compared to the other groups. Whereas there is high consistency between the correlations with resistance in the IAV-only and the IAV/SP-superinfection groups (Pearson’s *r* = 0.63, *P* ≪ 10^−10^, for a correlation across all genes; Fig. 4C), we also observe reprogramming of specific genes. For instance, the antiviral *Irf7* gene has a positive correlation with resistance in IAV-infected mice (Pearson’s *r* = 0.93) but a negative correlation with resistance in superinfection (Pearson’s *r* = – 0.42), suggesting “reprogramming” from positive to negative associations of *Irf7* with the resistance program (Fig. 4D). We further exemplify the inverse reprogramming with *Acads,* an essential gene that catalyzes an initial step of the mitochondrial fatty acid beta-oxidation pathway (Fig. 4D). Thus, during superinfection, and compared to IAV-only infection, *Irf7* is reprogrammed from induction to repression, whereas *Acads* is reprogrammed from repression to induction. The top 150 genes that are reprogrammed from induction to repression in the above comparison (highlighted in purple in Fig. 4C) are enriched with interferon-signaling genes (HG test *P* < 10^−22^, Fig. 4E; e.g., *Stat2, Irf7, Lgals3bp, Adar, Irf9,* and *Pml*). In contrast, the top 150 genes that are switched from repression to induction by resistance (highlighted in green in Fig. 4C) are enriched with metabolism-related pathways—for instance, adipogenesis and fatty acid metabolism (HG test *P* < 10^−6^ and 10^−8^, respectively, Fig. 4E; e.g., *Sod1, Hadh, Acads, Acaa1b, Adh7, Acaa2,* and *Hmgcs2*).

The analysis of resistance reprogramming during superinfection has several implications. First, it suggests the reuse of the existing resistance program (for IAV infection) in superinfection rather than the emergence of a new superinfection-specific program. Second, the analysis suggests metabolic reprogramming as a novel potential process that is involved in superinfection. Third, despite the overactivation of resistance in superinfection, we found that interferon signaling is not overactivated but rather is repressed with increasing resistance levels. We note that both the induction of metabolic reprogramming and the repression of antiviral response have not been previously reported, likely because these effects could only be discerned when considering gene expression relative to the background level of resistance. An important aspect needs to be underscored at this point. While we observe a tissue-level reprogramming of the resistance program, this does not exclude the possibility of reprogramming due to a change in cell composition. For example, while the reprogramming could be due to a change in the relative levels of interferon signaling and resistance at the intracellular level, it can also be envisaged that reprogramming is due to the dynamic change in cell composition, e.g., an increase in the abundance of cell types in which the resistance-to-interferon-signaling ratio is low. Indeed, we observe a substantial difference in cell composition of superinfection compared to a single infection (Fig. S5; Table S3). Even if the reprogramming of resistance is at the tissue level, this may still be a driver of susceptibility and an important therapeutic target. Future studies are needed to define the reprogramming of resistance at the single-cell-type and single-cell levels.

## DISCUSSION

Secondary bacterial infections are the most frequent complications during IAV pandemic outbreaks, contributing to excessive morbidity and mortality in the human population (27). Most IAV-related deaths are attributed to SP infections, which usually begin within the first week of IAV infection in the respiratory tracts (28, 29).

Many previous studies were set to explain the enhanced susceptibility to secondary bacterial infections. Early investigations have pointed to lung epithelium damage as a factor in enhanced bacterial adherence and growth (8, 10, 11). Other studies have shown that primary IAV infections impair macrophages and neutrophils’ functions in the lung (12, 13). Recent reports attributed various changes in cytokine levels, such as TNF-α, IL-6, type I IFN, IL-10, and IL-27 (16, 30–33), as well as changes in immune cells’ composition [e.g., monocytes, T cells, and B cells (17, 18, 34, 35)], which occur during the viral infection and in turn limit the innate pulmonary host defense against the subsequent bacterial invasion. Although these studies have greatly extended our understanding regarding the interactions of IAV and SP with the host response, these studies did not account for the immune state of the resistance to pathogens in each individual. Thus, the potential reprogramming of the key antimicrobial program (resistance) has not been investigated.

Our longitudinal data allowed fine analysis of gene expression changes during superinfection. For instance, we found that superinfected mice exhibited rapid gene expression induction or reduction within the first 12 h after encountering the second pathogen. Cell proliferation and immune response activation processes were upregulated, while endothelial processes, vasculogenesis, and angiogenesis were downregulated. There is a reduced level of several angiogenic growth factors, such as VEGF and Apln, which typically induce pro‐angiogenic pathways in endothelial cells (36, 37). These findings support the notion that in addition to the well-characterized impairment of the innate immune response, the downregulated endothelial signature may reflect vascular destruction, which in turn leads to enhanced bacterial dissemination (24).

Our study suggests a superinfection-specific reprogramming of the resistance program. First, despite the overall hyperactivation of resistance against the pathogen, the specific resistance response to superinfection also involves interferon signaling reprogramming (from a positive to a negative association with resistance). Furthermore, it is suggested that part of the resistance program—the fatty acid metabolism—is reprogrammed during superinfection from a negative to a positive association with resistance. The reprogrammed responses have not been previously reported, likely because these could only be revealed when considering the background state of the host defense. The reprogramming of resistance uncovers a switch in functional activities that likely involves intracellular changes (a molecular change relative to the intracellular resistance level), cellular changes (a change in cell-type composition), or both. Our study provides a starting point for investigating the drivers of the reprogrammed response. The reprogrammed functions (fatty acid metabolism and interferon signaling) are compelling new targets for switching the pathogenic superinfection state into a single-infection state, either at the cell level or the whole-tissue functionality. Overall, superinfection-specific reprogramming provides promising targets for future therapeutic interventions.

## MATERIALS AND METHODS

### Mice

Adult female C57BL/6 mice (Envigo, Israel) were housed on hardwood chip bedding under a 12-h light/dark cycle and humidity and temperature-controlled specific pathogen-free conditions at the animal facility of Tel Aviv University. Mice were given tap water and a standard rodent chow diet *ad libitum* from their weaning day until the end of the experiment.

### Treatment groups

The mouse-adapted H1N1 influenza A/PR/8/34 virus was grown in allantoic fluid of 10-day-old embryonated chicken eggs at 37°C for 72 h. Allantoic fluid was harvested, and viral titers were determined by standard plaque assay in Madin–Darby canine kidney (MDCK) cells (38). The virus was then stored at −80°C. SP (ATCC 6303, *S. pneumoniae* type 3 encapsulated strain) was grown for 16 h at 37°C in 5% CO_2_ on Columbia Agar plates supplemented with 5% (vol/vol) sheep blood. Colonies were picked and grown in Todd Hewitt broth with yeast extract to the mid-logarithmic phase, harvested, and diluted to concentrations of 1 × 10^4^ colony-forming units (CFU) (verified by re-plating 10-fold dilutions). Mice were subjected to four treatments (Table S1): control (mock) (PBS at time point 0 and 5 days), IAV-only infection (IAV at time point 0 and PBS at time point 5 days), SP-only infection (PBS at time point 0 and SP at time point 5 days), and IAV/SP superinfection (IAV at time point 0 and SP at time point 5 days). Mice were anesthetized intraperitoneally with a ketamine/xylazine cocktail. For IAV, anesthetized mice were administered intra-nasally with IAV (100 plaque-forming units [PFU], tittered on MDCK cells) at day 0. For SP, mice were infected intranasally with SP (1 × 10^4^ CFU) at day 5 post-Treatment 1. Mice were monitored for an additional 44 h after Treatment 2 (in total, 6 days and 20 h post-Treatment 1). Weight and survival were monitored daily. Mice that exhibited severe clinical signs of disease or mice with more than 20% weight loss were humanely euthanized.

### RNA isolation, library preparation, and sequencing

Lung tissues were collected into RNAlater (Qiagen) and lysed with QIAzol (Qiagen). RNA samples were isolated with miRNeasy kit (Qiagen), and their RNA Integrity Numbers were verified to be higher than 8 using the Agilent 2100 Bioanalyzer. cDNA libraries were prepared using 2 µg of the isolated RNA and the SENSE mRNA-Seq Library Prep Kit V2 for Illumina (Lexogen). DNA size and quality were checked using the Agilent 2100 Bioanalyzer. Libraries were quantified using the Qubit DNA HS Assay kit (Invitrogen). The amplified libraries, each sample with a unique index primer, were pooled at a total concentration of 2 nM and sequenced using the Illumina HiSeq platform (Technion Genome Center, Israel). Clustering, de-multiplexing, and alignment were performed as previously described (39). For the alignment, we applied a joint alignment of reads for the mouse genome and the IAV genome (viral transcripts NC_002016, NC_002017, NC_002018, NC_002019, NC_002020, NC_002021, NC_002022, and NC_002023). Raw counts were derived with featureCounts (40). Raw read counts were imported into R studio (version 3.6.1), normalized using the DESeq2 package (41)), and the log2 of the normalized levels are reported as gene expression levels. The total number of genes is 23,829. This data set is deposited in the GEO database (GSE206534).

### Detecting differentially expressed genes

Differential expressions were calculated using the DESeq function in the DESeq2 package (Wald test). We compared responses between experimental groups, requiring all samples in each group to be during the acute phase of infection (i.e., a high pathogen load), whenever possible: (i) superinfection—high pathogen load in all selected samples: time point > 5 days, *n* = 16. (ii) IAV-only—high pathogen load in all selected samples: log_2_ viral mRNA > 2 at 2–6 days post-Treatment 1 (Fig. 1D), *n* = 14; (iii) SP-only and controls—no pathogen detected in all samples; therefore, we used all samples, *n* = 7 and 10, respectively. Reported DEGs for each comparison obtained *q* values (i.e., FDR-adjusted *P*-values) that are lower than 0.05 and FC levels that are higher than 1.5 (either for up- or downregulation) (see IAV-only DEGs, SP-only DEGs, superinfection vs IAV-only DEGs, and superinfection vs SP-only DEGs in Table S2). Superinfection DEGs are defined as in Fig. 2E, and the “top superinfection DEGs” are superinfection DEGs whose *q* < 10^−5^ for the comparison between superinfection and IAV-only groups (Table S2).

The DESq function pools the samples from each group and ignores the time point of each sample. Due to this simplifying assumption, we calculated additional alternative scores of more fine-grained time intervals: (i) only time points 3–4 days of the IAV-only group (*n* = 5), (ii) only time points 5–6 days of the IAV-only group (*n* = 7), (iii) only early time points (time points 5–5.5 days post-Treatment 1) of the superinfection group (*n* = 7), and (iv) only late time points (time points 5.5–7 days post-Treatment 1) of the superinfection group (*n* = 7). Overall, we saw a substantial overlap between the alternatives (e.g., an average of *R*^2^ = 80% and a minimum of *R*^2^ = 70% between any pair of alternatives for the log *q*-values of the superinfection vs IAV-only comparison).

### Bacterial load quantification in lungs

For bacterial load quantification (by 16S rRNA measurements), total RNA (1 µg) was reverse transcribed using the SuperScript kit (BioRad), and the real-time PCR was performed in three technical replicates using the Applied Biosystems StepOnePlus Real-Time PCR system and Fast SYBR Green Master Mix (Applied Biosystems). Oligonucleotides for 16S rRNA were

16s-F: GGTGAGTAACGCGTAGGTAA and 16s-R: ACGATCCGAAAACCTTCTTC.

Relative gene expression levels of the 16S rRNA were normalized relative to the control (mock-infected) samples.

Of note, since we processed/lysed the lung tissues in RNAlater/QIAzol—a methodology incompatible with maintaining bacteria viability—we could not use a culture-based analysis.

### IAV load quantification in lungs

Our RNA-seq library preparation captured both cellular and viral mRNA (positive-sense, polyadenylated viral RNA). IAV load was measured using the viral mRNA levels in the lung tissue based on the RNA-seq measurements. As detailed above, we aligned the RNA-seq reads of all samples against the mouse and the IAV genomes and quantified the viral gene expression. The reported viral load is the average of (log2-scaled) viral mRNA across all IAV genes.

Of note, since the cellular replication of IAV is characterized by the synthesis of polyadenylated mRNA molecules with positive polarity, but free IAV virions encapsidate the non-polyadenylated viral genome with a negative polarity, the quantification of the viral polyadenylated mRNAs reflects intracellular viral replication rather than free virions.

### Statistical analysis

Enrichment analyses were performed using functional classes in the MSigDB’s hallmark collection (Fig. 4E), Gene ontology, the “Biological process” collection (Fig. 3B), and the Ingenuity Pathway Analysis (Fig. 3D). Multiple testing FDR correction was applied in all cases.

We performed a comparison of trends over selected time points (e.g., Fig. 1B through D, Fig. 4B) using the “Chow test,” which analyzes the statistical difference between experimental groups in their response over time. The Chow test assumes a linear regression and tests whether the model (intercept or slope of change over time) in one experimental group differs from the model in the other group. As the Chow test does not require repeats in each time point and does not require that the selected time points be the same in the two groups, it fits the experimental design in this study. Of note, we used a different test to identify differentially expressed genes (see above), because we compared different time intervals based on their similar pathogen-load characteristic.

### Evaluation of cell quantities

Cell quantities (Fig. S5; Table S3) were inferred from gene expression profiles using a deconvolution approach—either CIBERTSORT (42) or CPM (43). Reference profiles for calculating the cell quantities were either from Steuerman et al. (44) or the Tabula Muris collection (45).

### The resistance model

#### Background

We used a previous model of the host response to infection (20). In this previous study, two host-defense transcriptional programs, termed “disease-tolerance” and “resistance,” were formulated and characterized. The analysis by Cohn et al. (20) was performed in four steps. As a first step, the programs were defined by dimension reduction of lung transcriptome data in response to IAV infection, based on the variation in different time points and across 33 different Collaborative-Cross murine strains (20). This dimension reduction revealed two dimensions that together explain a large fraction of the variation in the IAV-infected murine lungs. Each of these dimensions is referred to as a “program.” For each of these programs, the dimension reduction associated each gene with a certain “gene weight.” Thus, each program was defined by a vector of gene weights across all genes. The second step was to devise a method for calculating the personal states, or “levels,” of each program based on a given transcription profile and the predefined gene weights of the two programs. The formulation of this calculation is detailed below (Equation 1). The third step was to interpret the biological meaning of each program. To that end, each program’s levels were compared to clinical and physiological characteristics during the course of infection, as well as prior functional knowledge about the high-weight genes of each program. This analysis indicated that (i) one program was primarily related to the physiological and molecular maintenance of healthy tissue in the presence of pathogens. This program was therefore referred to as the “disease-tolerance” program; (ii) the other program was found to be related to the ability to eliminate the pathogen. Thus, this program was termed the antimicrobial “resistance” program. For instance, it was shown that the levels of the resistance program (but not the disease-tolerance program) are linked to the IAV load in the lungs; the resistance levels are upregulated in various viral and bacterial infections; the levels of the disease-tolerance program are linked to both abiotic and biotic stress, whereas the levels of the resistance program are linked only to biotic (rather than abiotic) stress. Together, these findings highlighted a pathogen-specific induction of the resistance program following a broad spectrum of pathogens (20). Finally, the fourth step was to investigate the programs across cell types. Analysis of scRNA-seq data has shown that the identified programs are part of a generic molecular response in 28 immune cell types, as well as human bronchial epithelial cells (20).

#### Calculation of the resistance levels

Resistance levels were calculated by a predefined linear combination of all genes (20). Gene weights were previously calculated for 14,380 of the 23,829 genes in the current study; these gene weights were used for the calculation of resistance levels and are provided in Table S4. The calculation of the resistance state takes into consideration the status of disease tolerance as an additional covariate: for a given individual i, the resistance level is defined using a linear model that combines genome-wide expression profiling with prior knowledge of the weights of each gene,


(1) $$Z_{i}=b_{i}+s_{R}^{i}V_{R}+s_{T}^{i}V_{T}$$

Here, $Z_{i}$ is the vector of all relative measured gene expression levels of individual i, $V_{R}$ is the predefined vector of gene weights across all genes for the resistance program (Table S4), and $s_{R}^{i}$ indicates the output “resistance level” (“resistance state”) of individual i. To account for confounding variation in disease tolerance, this formulation also includes $V_{T}$ , the predefined vector of gene weights for the disease-tolerance program (Table S4), and $s_{T}^{i}$ , which is the output “disease-tolerance level” of individual i. Of note, before calculating the resistance levels ($s_{R}^{i}$), the expression levels of each gene were centered according to the measurements in the control (mock-infected) mice.

#### Exclusion of outliers

Given the joint inference of resistance and disease tolerance, we marked as an outlier any sample that is farther than two standard deviations from the best-fit resistance/tolerance line. Three samples were excluded as outliers from the analysis of resistance based on this approach (Fig. S4; Table S1).

#### Evaluation of the resistance program

We used two scores to assess the quality of the resistance model. (i) The percentage of explained inter-individual variation. For each gene *g*, we calculated the percentage of variation that is explained by resistance. We calculated the regression between the resistance levels (dependent variable) and the measured gene expression levels of gene *g* (independent variable) across individuals. The “percentage of explained inter-individual variation” is the *R*^2^ of this regression. The same analysis was applied independently to each of the experimental groups. For comparison, this calculation was applied to permuted data (reshuffling the expression levels of each gene *g*). The comparisons of *R*^2^ values from real and permuted data were used to calculate the empirical *P*-value for each gene *g*. For each experimental group separately, we report the distribution of the percentage of explained inter-individual variation across genes (Fig. S3A, left) and the percentage of genes with empirical *P*-value < 0.05 based on the comparison with permuted data (Fig. S3A). For example, for superinfection, 20.6% of all measured genes obtained empirical *P*-value < 0.05 (Fig. S3A). Overall, we saw that a substantial fraction of the genes obtained empirical *P*-value < 0.05 in all experimental groups, including the superinfection group (16.2%, 15.4%, 34.6%, and 20.6% for controls, SP-only, IAV-only, and superinfection groups, respectively). (ii) The percentage of explained inter-gene variation. To assess whether the predefined genes’ weights (Table S4) are indeed relevant to superinfection, we used the ability of these weights to predict the variation between genes within each individual subject. The analysis was performed separately for each gene expression profile. Relying on (Equation 1) and using a given individual *i*, we calculated regression between the weights in $V_{R}$ and $V_{T}$ (independent variables) and the expression profile of all genes in individual *i* (dependent variable). We used the *R*^2^ of the regression for individual *i* as the evaluation metric, which is the percentage of explained inter-gene variation for individual *i*. For comparison, this calculation was applied to permuted data (reshuffling the expression levels of genes in each individual *i*). For each experimental group separately, we report the distribution of the percentage of explained inter-gene variation across individuals (Fig. S3B, left) and the percentage of individuals with empirical *P*-value < 0.05 based on the comparison with permuted data (Fig. S3B, right). All individuals in all experimental groups obtained empirical *P*-value < 0.05 with respect to the inter-gene variation that is explained by the model (Fig. S3B), supporting the validity of the model for superinfection.

## SUPPLEMENTAL MATERIAL

Supplemental material msystems.01048-23-s0001.pdf Figures S1 to S5 and captions to Tables S1 to S4.

Supplemental Tables msystems.01048-23-s0002.xlsx Tables S1 to S4.

ASM does not own the copyrights to Supplemental Material that may be linked to, or accessed through, an article. The authors have granted ASM a non-exclusive, world-wide license to publish the Supplemental Material files. Please contact the corresponding author directly for reuse.
