# Supplementary material for: The host transcriptional response to superinfection by influenza A virus and Streptococcus pneumoniae
Source: mSystems. 2024 Mar 6;9(4):e01048-23. doi: 10.1128/msystems.01048-23 (PMC11019783; doi:10.1128/msystems.01048-23)

**Supplementary Material**

# The host transcriptional response to superinfection by influenza A virus and *Streptococcus pneumonia*

## Supplementary Figures Legends

**Supp Figure 1: Analysis of response**.

(**A**) **PCA analysis**. **Left**: A scatter plot of PC1 (y-axis) against the time point (x-axis) of each sample (a circle). The PC1 values are as in **Figure 2A**. The four treatment groups are color-coded. **Right**: Comparison of PC1 values between treatment groups. Chow test was performed using all samples after Treatment 1: $n$=16, 7, 7, and 5 for the superinfection, IAV-only, SP-only and control groups, respectively (**Supp. Table 1**).

(**B**) **Response to IAV treatment**. For each gene, the scatter plot presents the effect sizes of two comparisons. X-axis: log2 fold change of comparison between IAV-only and control treatments – namely, the response to IAV treatment without subsequent bacterial treatment. Y-axis: log2 fold change of comparison between superinfection (IAV+SP) and SP-only treatments – namely, the response to IAV treatment, with the presence of a subsequent bacterial treatment. The plot shows high consistency (linear regression's $R^2$=0.28, $p \ll 10^{-100}$), indicating reproducibility of the IAV-effect in two distinct comparisons.

(**C**) **Response to SP treatment**. **Left**: For each gene, the scatter plot compares the effect sizes of two single-infection treatments. X-axis: log2 fold change of comparison between IAV-only and control treatments. Y-axis: log2 fold change of comparison between SP-only and control treatments. The plot shows high consistency (linear regression's $R^2$=0.19, $p \ll 10^{-100}$). **Right**: Paired t-test between the effect sizes (log2 fold change) of two comparisons. In all cases, the log2 FC values of the SP-only-vs-control comparison were significantly lower than the log2 FC values of the other comparisons. The comparison to the IAV-only-vs-control is exemplified in the left panel.

**Supp Figure 2: Genome-wide expression profiles**. Gene expression profiles during response to treatments. Included are 1500 genes with the highest variance across all 51 samples. Shown are the relative gene expression levels for each gene (blue to red scale in each row). The genes are partitioned into four clusters using *k*-means clustering (indicated on the right). Clusters are ranked by the cluster size. Samples are ordered by time points (left to right) within each of the four experimental groups (increasing time points are indicated as black triangles).

**Supp Figure 3: Assessing resistance based on inter-gene and inter-individual variation**. The analysis was applied to all samples (before excluding outliers), i.e., $n$=18, 16, 10, 7 for the IAV-only, superinfection, control and SP-only groups, respectively.

(**A**) **Inter-individual variation analysis**. **Left**: For each gene and each experimental group, we calculated the percentages of inter-individual variation that is explained by the resistance level. Each box plot describes the distribution of these calculated percentages across all genes (y-axis), calculated for a specific experimental group (x-axis). Gray: distributions calculated using the measured data. The red dot indicates the top 5% genes with the highest percentages of explained inter-individual variation when calculated using permuted data (10,000 repeats). In other words, the red dot indicates an "empirical p-value" cutoff of 0.05. The percentage of genes above this cutoff are indicated in the summary table (right panel). **Right**: summary table. For each experimental group, the table reports the percentage of genes that obtained empirical p–value < 0.05 with respect to their inter-individual variation that is explained by the model. The analysis shows that the resistance level captures a substantial fraction of the variation among individuals, not only during IAV infection but also during superinfection.

(**B**) **Inter-gene variation analysis**. **Left**: For each individual and each experimental group, we calculated the percentages of inter-gene variation that is explained by the model (Eq. 1). Each box plot describes the distribution of these percentages across all individuals (y-axis), calculated for different experimental groups (x-axis). Gray: distributions calculated using the measured data. The red dot indicates the top 5% of individuals with the highest percentages of explained inter-gene variation when calculated using permuted data (10,000

repeats). In other words, the red dot indicates an 'empirical p-value' cutoff of 0.05. The percentage of individuals above this cutoff are indicated in the right panel. **Right:** summary table. For each experimental group (row), the table reports the median of the percentage of explained inter-gene variation (column 1) and the percentage of individuals that obtained empirical p–value < 0.05 (column 2). The analysis shows that the model in Eq. 1 captures a substantial fraction of the variation among genes within each individual, not only during IAV infection but also during superinfection.

**Supp Figure 4: Resistance levels.** The scatter plots present the inferred disease-tolerance level (x-axis) and resistance level (y-axis) of each individual mouse (a dot), either for the IAV-only treatment (left) or the IAV/SP superinfection treatment (right). Individual samples located farther than two standard deviations from the best-fit line are indicated in orange and were excluded from all analyses in **Figure 4** (see **Supp. Table 1**, **Methods**).

**Supp Figure 5. Cell type abundance.** The scatter plots provide the inferred quantity of each cell type (plots, indicated on top) in each time point (x-axis). Each sample is represented by a circle, color-coded by the experimental group. The relative abundance levels were estimated using the CIBERSORTx algorithm. The reference data used to infer cell type quantities is indicated on top (Top: Tabula Muris (Schaum et al., 2018), Bottom: Steuerman et al., 2018). **Supp. Table 3** reports the inferred cell-type quantities using different methods and different reference profiles, for all four experimental groups.

## Supplamantry Tables Legends*

*All tables are in a separate data file

**Supplementary Table 1. Mice under study.**

**Supplementary Table 2. Differentially expressed genes.** In columns 1-13, for each of the comparisons between treatment groups (indicated in row 1), reported are the differential expression $q$ values (minus log 10), log2 fold change, and indications of DEGs (indicated in row 2). Superinfection-DEGs are reported in column 14. The top superinfection-DEGs are reported in column 15, selected based on the $q$-values for the superinfection (n=16) vs. IAV-only (n=14) comparison in column 8.

**Supplementary Table 3. Cell type abundance.** The table reports, for each sample (columns 1,2) in each experimental group (column 3), its inferred cell-type abundance (columns 4-33) using different algorithms and reference datasets (indicated on the top line). The cell types are indicated in line 2.

**Supplementary Table 4. Gene weights for the calculation of resistance and disease tolerance levels.** All resistance levels inferred in this study were calculated with these weights using Eq. 1 (**Methods**). The gene weights are from Cohn *et al.,* 2022.

# Supp Figure 1

**A**

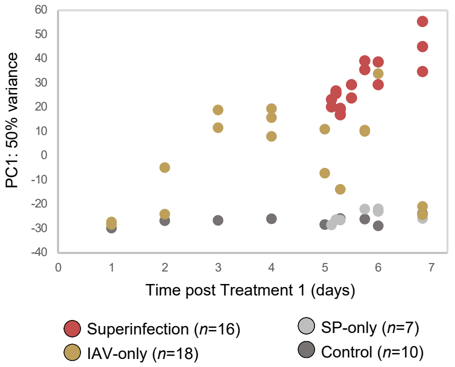


| Compared groups | Chow p-value |
|---|---|
| Superinfection (16) vs. IAV-only (7) | 0.002 |
| Superinfection (16) vs. SP-only (7) | $10^{-11}$ |
| Superinfection (16) vs. control (5) | $10^{-14}$ |

**B**

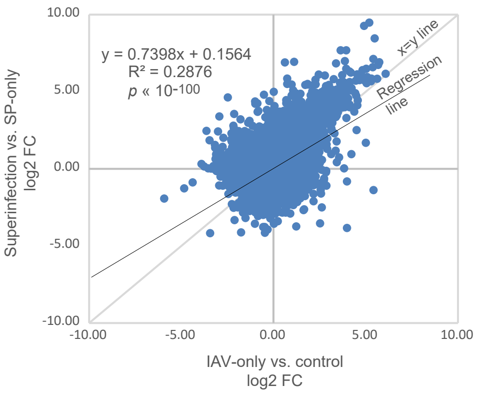


**C**

Paired t-test p < $10^{-138}$

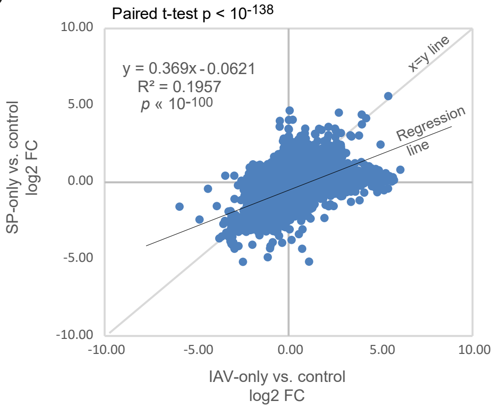


| **Comparison 1** | **Comparison 2** | **Paired t-test *p*** |
|---|---|---|
| IAV-only vs. control | SP vs. control | $10^{-138}$ |
| Superinfection vs. SP-only | SP vs. control | $10^{-150}$ |
| Superinfection vs. IAV-only | SP vs. control | $10^{-163}$ |

## Supp Figure 2

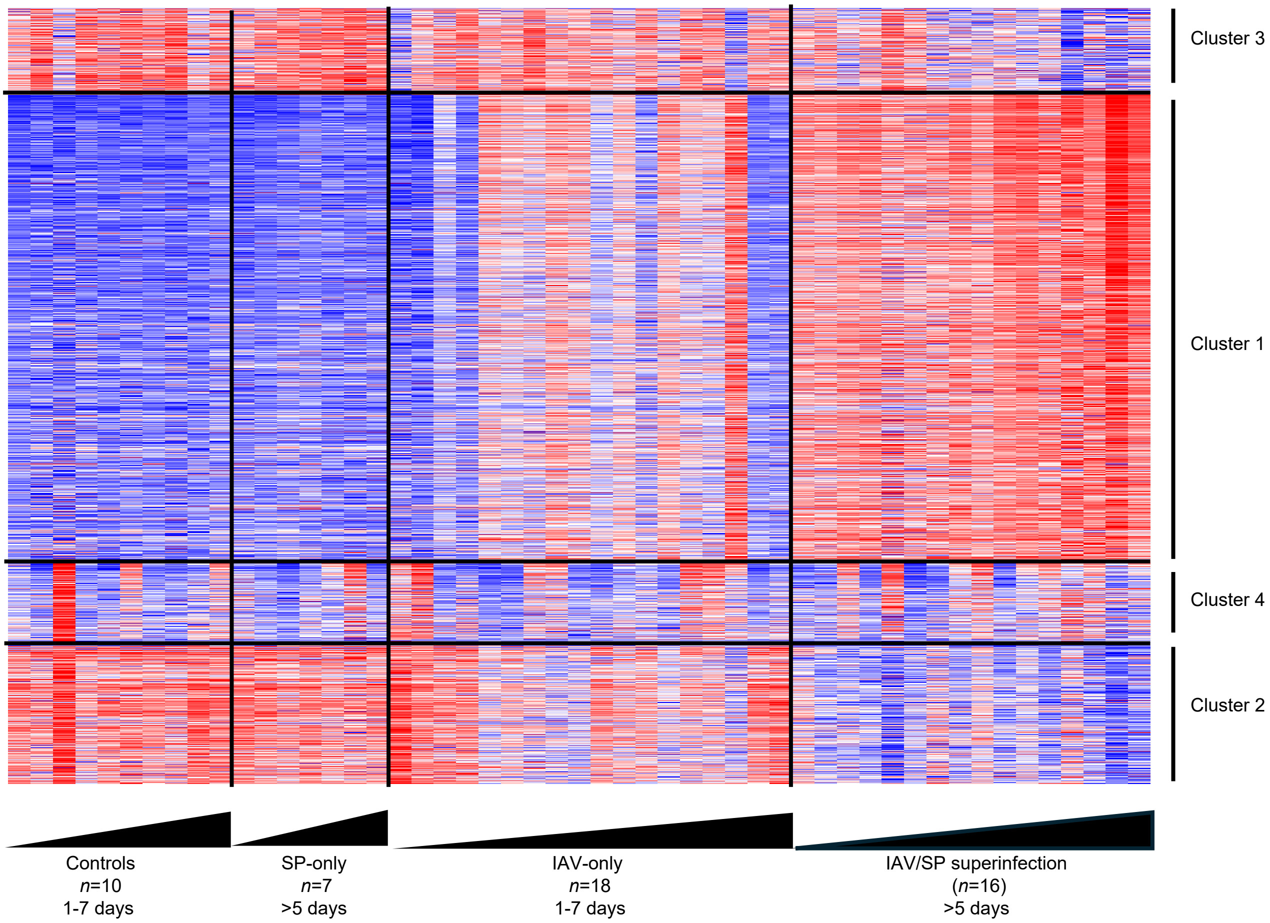

## Supp Figure 3

**A**

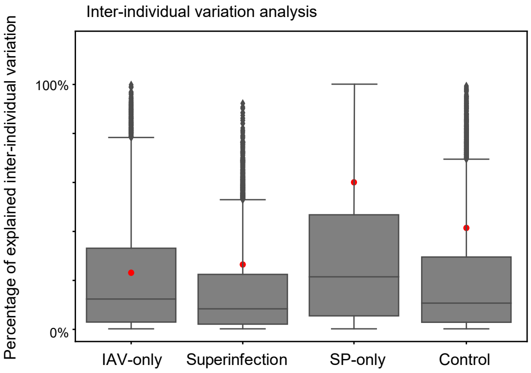


| Experimental group | % genes with significant 'percentage of explained inter-individual variation' |
|---|---|
| Control group | 16.2% of genes* |
| SP-only group | 15.4% of genes* |
| IVA-only group | 34.6% of genes* |
| Superinfection group | 20.6% of genes* |

*Out of 14,380 genes

**B**

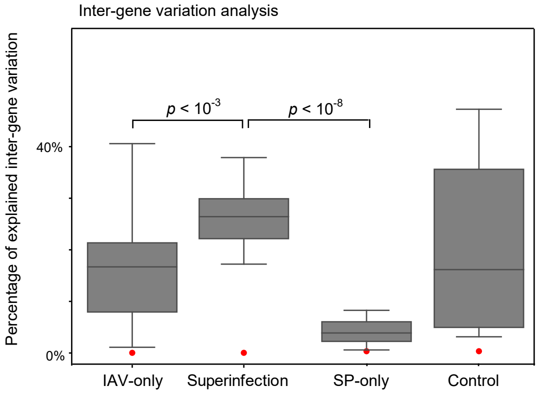


| Experimental group | Median 'percentage of explained inter-gene variation' | % individuals with significant 'percentage of explained inter-gene variation' |
|---|---|---|
| Control group | 16% | 100% of individuals |
| SP-only group | 4% | 100% of individuals |
| IVA-only group | 17% | 100% of individuals |
| Superinfection group | 26% | 100% of individuals |

## Supp Figure 4

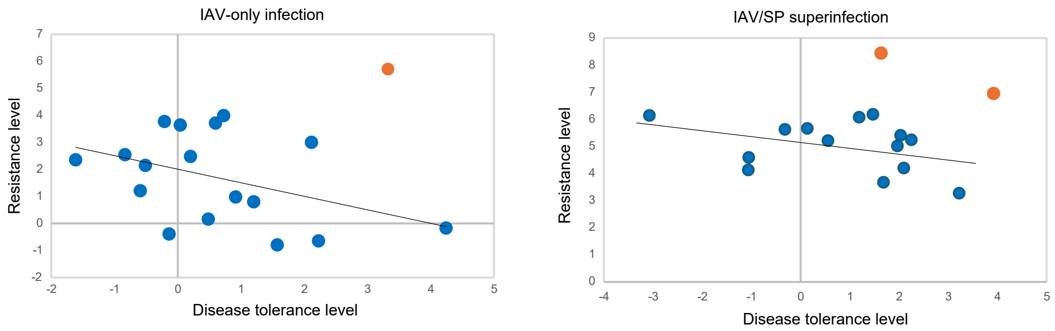

## Supp Figure 5

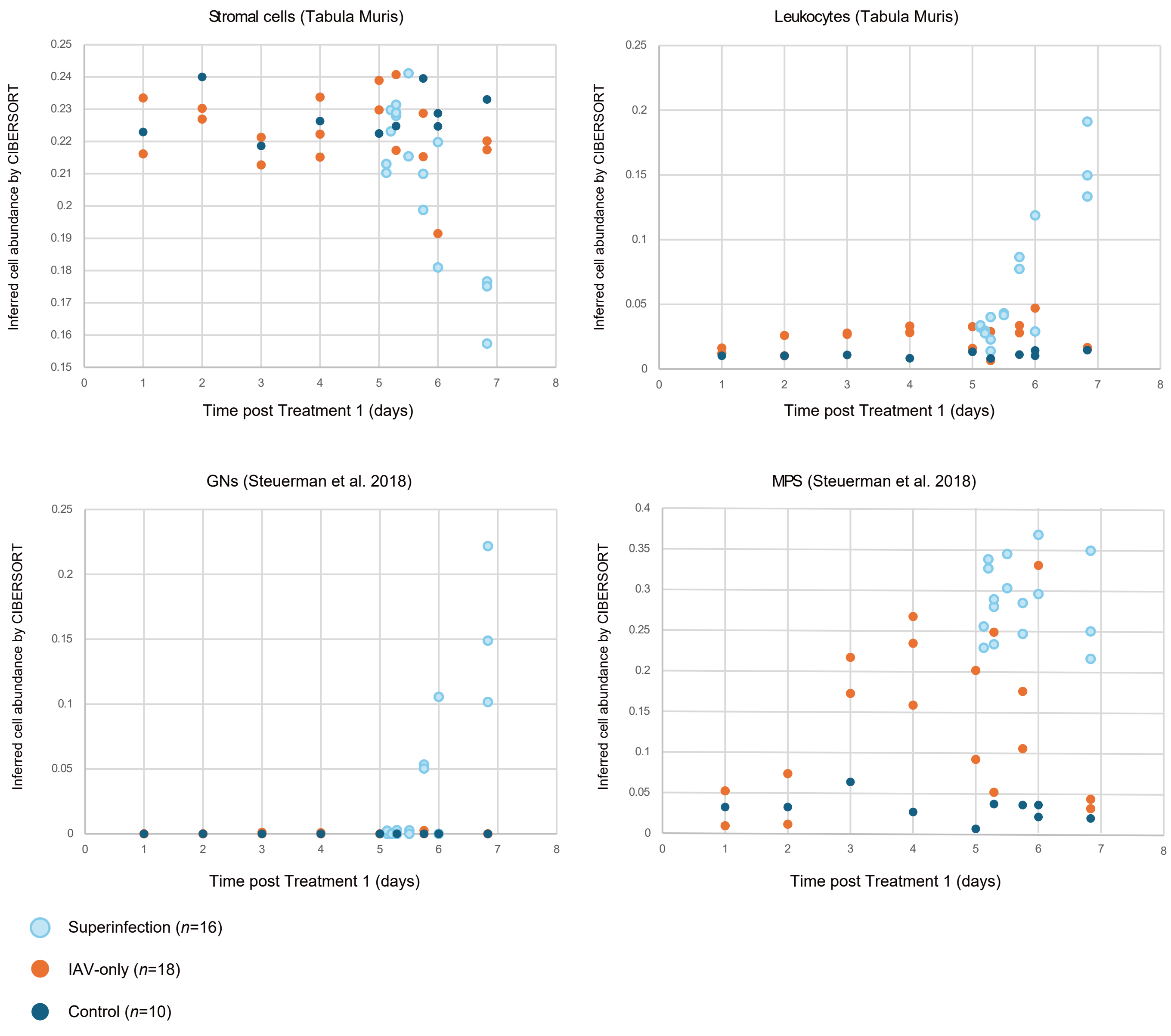

Supplement: Supplemental material — Figures S1 to S5 and captions to Tables S1 to S4. [file msystems.01048-23-s0001.pdf]
